# Vestibular Activation Habituates the Vasovagal Response in the Rat

**DOI:** 10.3389/fneur.2017.00083

**Published:** 2017-03-15

**Authors:** Bernard Cohen, Giorgio P. Martinelli, Yongqing Xiang, Theodore Raphan, Sergei B. Yakushin

**Affiliations:** ^1^Department of Neurology, Icahn School of Medicine at Mount Sinai, New York, NY, USA; ^2^Department of Computer and Information Science, Brooklyn College, City University of New York, New York, NY, USA

**Keywords:** baroreflex sensitivity, head-up tilt, sinusoidal galvanic vestibular stimulation, vestibulosympathetic reflex, vasovagal syncope

## Abstract

Vasovagal syncope is a significant medical problem without effective therapy, postulated to be related to a collapse of baroreflex function. While some studies have shown that repeated static tilts can block vasovagal syncope, this was not found in other studies. Using anesthetized, male Long–Evans rats that were highly susceptible to generation of vasovagal responses, we found that repeated activation of the vestibulosympathetic reflex (VSR) with ±2 and ±3 mA, 0.025 Hz sinusoidal galvanic vestibular stimulation (sGVS) caused incremental changes in blood pressure (BP) and heart rate (HR) that blocked further generation of vasovagal responses. Initially, BP and HR fell ≈20–50 mmHg and ≈20–50 beats/min (bpm) into a vasovagal response when stimulated with Sgv\S in susceptible rats. As the rats were continually stimulated, HR initially rose to counteract the fall in BP; then the increase in HR became more substantial and long lasting, effectively opposing the fall in BP. Finally, the vestibular stimuli simply caused an increase in BP, the normal sequence following activation of the VSR. Concurrently, habituation caused disappearance of the low-frequency (0.025 and 0.05 Hz) oscillations in BP and HR that must be present when vasovagal responses are induced. Habituation also produced significant increases in baroreflex sensitivity (*p* < 0.001). Thus, repeated low-frequency activation of the VSR resulted in a reduction and loss of susceptibility to development of vasovagal responses in rats that were previously highly susceptible. We posit that reactivation of the baroreflex, which is depressed by anesthesia and the disappearance of low-frequency oscillations in BP and HR are likely to be critically involved in producing resistance to the development of vasovagal responses. SGVS has been widely used to activate muscle sympathetic nerve activity in humans and is safe and well tolerated. Potentially, it could be used to produce similar habituation of vasovagal syncope in humans.

## Definitions

Habituation: “A behavioral response decrement that results from repeated stimulation and that does not involve sensory adaptation/sensory fatigue or motor fatigue” (1, 2). Susceptible Rats: rats that readily had vasovagal responses from vestibular (otolith) stimulation. Vasovagal Response: a combined fall in blood pressure (BP) of ≈20–50 mmHg and ≈20–50 bpm that takes seconds to develop and is followed by a slower return to baseline levels over the course of minutes. Vasovagal Syncope: a loss of consciousness due to a decrease in BP and heart rate (HR) causing a loss of blood flow to the brain. Syncope: fainting, loss of consciousness—old English, without cause.

## Introduction

Vasovagal syncope is a significant medical problem (3–6). The symptoms that lead to vasovagal syncope and the preceding vasovagal response that underlies syncope have been well described (5, 7, 8), and the reductions in blood pressure (BP), heart rate (HR), and baroreflex sensitivity associated with syncope are also known (7, 9–11). Using combined tilt and lower body negative pressure, as BP fell, HR transiently increased but then also collapsed in the pre-syncopal state (7). A critical observation was that the vasovagal response and vasovagal syncope involved a reduction in baroreflex sensitivity (7, 10, 12, 13). As yet, neither is it known why this occurs nor is it apparent where and what the signal might be that initiates the combined fall in BP and HR. A model of the vasovagal response has suggested, however, that a fall in desired BP could be the critical signal that initiates the combined fall in BP and HR (14). Where such a signal would arise and how it is transmitted is still unknown. Consequently, there has been no effective therapy for vasovagal syncope.

Therapeutic measures for unexpected syncope have included beta blockers, corticosteroids, and pacemakers (5, 8, 15), but none of these has been generally more effective than placebo (8, 16). An apparently promising therapy in which “syncope-sensitive” patients were repetitively tilted 60° for prolonged periods was originally shown to habituate the vestibulosympathetic reflex (VSR) and to reduce or abolish syncope (17–21). These studies used repeated episodes of 60° static head-up tilt or standing against a wall for substantial periods of time that activated otolith and body tilt receptors (22–24), both of which play a role in producing cardiovascular changes through the VSR in humans (25–29). In fact, 60° head-up tilt is widely used to determine if humans suspected of having vasovagal syncope become faint during the tilt-test. Sustained habituation of syncope was not found in other studies that utilized tilt training (30, 31). Foglia-Manzillo speculated that while it was probably possible to habituate some subjects with prolonged bouts of static head-up tilt, the habituation techniques were too tedious to be effective in the general population (30). If a less tedious procedure that activated the vestibular system were to be devised, it could be used to habituate syncope through the VSR.

Recently, we developed a small animal model of the cardiovascular changes during vasovagal responses in the anesthetized rat (14, 32–35). In these studies, susceptible rats readily developed synchronous ≈20–50 mmHg decreases in BP and ≈20–50 bpm decreases in HR over seconds that recovered slowly over minutes in response to repeated vestibular (otolith) stimulation, i.e., to sGVS and 70° nose-up tilt. The sudden decreases in BP and HR and the slower return to pre-stimulus values are the essential components of the vasovagal response that underlie and generate vasovagal syncope (7, 9–11, 36). A wide range of vestibular stimuli that activate the otolith system are capable of inducing vasovagal responses (32–34). These include ±2 and ±3 mA, 0.025 and 0.05 Hz sinusoidal galvanic vestibular stimulation (sGVS), translation while rotating, ±70° oscillation in pitch, and 70° head-up tilt (32–34). Specific frequencies were critical for the generation of vasovagal responses, namely, vasovagal responses were only induced with low-frequency stimulations (0.025 and 0.05 Hz sGVS) and stimulus currents of ± 2 and ± 3 mA (33, 34). Moreover, vasovagal responses were only induced when there were 0.025 and 0.05 Hz oscillations in BP and HR (33, 34).

A striking finding in the previous experiments was that rats that were initially susceptible to the induction of vasovagal responses progressively lost their susceptibility as testing continued in the experiments that formed the basis for three papers that were previously reported (32–34). None of these rats had an increase in susceptibility when tested regularly; although, cessation of testing for prolonged periods could result in transient increases in susceptibility. However, when these animals were stimulated again, such increased sensitivity quickly disappeared. This loss of susceptibility to vasovagal responses suggests that the rats were becoming progressively habituated by the recurrent stimulation of the vestibular (otolith) system through activation of the VSR, i.e., they had met the classic criteria for habituation as defined by Rankin and Thompson: “Habituation is defined as a behavioral response decrement that results from repeated stimulation and that does not involve sensory adaptation/sensory fatigue or motor fatigue” (1, 2).

Thus, the first objective of the present study was to document the loss of sensitivity through a more detailed study of animals that were initially susceptible to the induction of vasovagal responses but became habituated when repeatedly given stimuli that excited the VSR. Additionally, we intended to show the various stages that were involved in the habituation, i.e., the specific changes in BP and HR that occurred as the rats became insensitive to development of vasovagal responses, since, to our knowledge, this has not been described before.

A second goal was to determine whether both ±2 and ±3 mA sGVS produced habituation of vasovagal responses. The ±2 mA sGVS has been widely used to study muscle sympathetic nerve activity (MSNA) in humans. The sGVS used to generate MSNA is safe and does not have significant side effects other than mild nausea in some subjects (37–40). Thus, if effective, ±2 mA sGVS could potentially be used to induce habituation and reduce susceptibility to vasovagal syncope in humans.

Since vasovagal responses and vasovagal syncope are associated with a reduction in baroreflex sensitivity (7, 10), the third goal of this study was to determine whether the habituation that had been observed as a result of vestibular stimulation was also associated with a change in baroreflex sensitivity. Finally, we intended to determine whether the low-frequency activity, which is critically associated with production of vasovagal responses, would be affected by the habituation process (33, 34, 36).

## Materials and Methods

### Animals

Eleven adult, male, Long–Evans rats (Harlan Laboratories, MA, USA) weighing between 400 and 500 g were used in this study. These rats were selected for their susceptibility to the development of vasovagal responses when stimulated with ±2 and ±3 mA, 0.025 Hz sGVS; 70° nose-up tilts (0.91 *g*); and ±70° oscillation in pitch (± 0.91 *g*). Results of experiments in seven of these rats have been reported in previous publications (14, 32–35). However, in these studies, we did not address the change in susceptibility produced by repeated vestibular stimulation. Four other rats (R009, R011, R012, and R020), that were also highly susceptible to the generation of vasovagal responses, were used in this study to characterize the changes in BP and HR associated with habituation of vasovagal responses. All experiments were approved by the Institutional Animal Care and Use Committee of the Icahn School of Medicine at Mount Sinai.

### Surgical Procedures, Implantation of a DSI Telemetric Sensor (St. Paul, MN, USA)

Surgery and testing were conducted under isoflurane anesthesia (4% induction, 2% maintenance with oxygen). Rats were prepared for aseptic surgery, and normothermia was maintained during and after surgery by keeping the animals on an Isotherm heating pad regulated by the feedback from a rectal thermometer. A telemetric BP sensor (DSI, St. Paul, MN, USA) was implanted in the abdominal aorta during aseptic surgery. The femoral artery, which was exposed through a small incision in the inguinal area, was dissected from the surrounding tissue, mobilized, and temporarily occluded. After dilating the vessel with a drop of lidocaine, the transmitter catheter was inserted into the vessel *via* a partial transverse arteriotomy. After releasing the occluding ligature, the catheter was gently threaded into the abdominal aorta and secured in place with two ligatures around the femoral artery. The body of the transmitter was placed in the flank of the rat in a subcutaneous pocket sealed with a purse-string suture. The skin was closed with surgical clips. Surgical pain was managed with pre-emptive and post-operative administration of Buprenorphine (0.05 mg/kg, SQ BID for 72 h). The animals were allowed a 7- to10-day recovery period before being used in experiments.

During experiments, the animals were anesthetized with isoflurane (4% induction, and 2% maintenance with oxygen) and kept on a heating pad (27, 28, 32–34). The sinusoidal galvanic vestibular stimulation (sGVS) was generated by a computer-controlled stimulator (32–34, 41, 42). Currents were delivered *via* sub-dermal needle electrodes placed in the skin over the mastoids. Rats were also subjected to a tilt-test, i.e., a 70° nose-up (0.91 *g*) change in position using a computer-controlled tilt table.

### Habituation Protocol

Four susceptible rats (R009, R011, R012, and R020) were studied intensively. After initial testing, they were trained for 5 days with recurrent sGVS over 2 weeks with three, 30 min epochs of ±2 mA, 0.025 Hz sGVS (R009) or ±3 mA, 0.025 Hz sGVS (R011, R012, and R020) on each day. The rats were in the prone position under anesthesia while they were receiving the sGVS. HR and BP were recorded, and the rats were tested with 70° head-up tilt before and after each period of habituation. Each of the four susceptible animals that were habituated had a vasovagal response to each stimulus when initially tested. Each experimental session began with a 45-min test period, followed by three alternating habituation and test periods. If there was no response to the head-up tilt, 15 min were allowed to elapse before the next habituation sequence began. If a vasovagal response was induced, 30 min were allowed to elapse before the next habituation sequence.

### Measurement of BP and HR

Intra-aortic BP telemetric data were continuously captured by a wand receiver (DSI, St. Paul, MN, USA) placed in close proximity to the animals, and BP and HR were recorded. The BP and HR, as well as the position of the tilt table and the current levels of sGVS were sampled at 1 kHz with 12 bit resolution (Data Translation, Inc., Marlborough, MA, USA). HR was computed offline from the systolic peaks in the BP. It was converted to an analog signal in beats per minute (bpm). BP was derived from systoles and diastoles, and mean BP was also calculated. When they were compared, there was no substantial difference between them. Therefore, the BP in this report is an estimate of averaged systolic BP, but reflects both mean and diastolic BP. Because measurements of BP were dependent on the occurrence of systoles, which occurred on average at about 300–330 bpm, the effective sampling rate for the BP derived from the systoles was limited to ≈1 sample/200 ms, i.e., 5 samples/s. Low-frequency oscillations in BP and HR (0.025 and 0.05 Hz), which are active during vasovagal responses (34) were also monitored (see Wavelet Analysis).

### Measurement of Baroreflex Sensitivity

Twenty seconds of BP signal before the onset of the sGVS were used to calculate the baroreflex sensitivity. A peak finding algorithm identified each systolic/diastolic cycle. The time durations between two systolic peaks (systBP_i_ and systBP_i_ + 1) or the RR-interval, were plotted against the first systolic peak (systBP_i_). The slope of the linear regression was defined as the baroreflex sensitivity, i.e., the ratio between the change of the RR-interval and the change of systolic BP.

### Phase Plots

The temporal sequence of the changes in BP and HR induced by sGVS from the beginning of the habituation process to its end were determined in a typical rat (R009). This animal, which was initially susceptible to generation of vasovagal responses, became progressively resistant as it became habituated. BP was plotted against HR in response to single trains of sGVS from the end of the train until the resting BP was reached. From this, it was possible to determine how the direction and magnitude of the modulations in BP and HR were altered during the habituation process. The data were fitted with a least squares regression, and the slopes were plotted sequentially from the beginning of testing until the end of habituation. This involved 33 tests over the 10 habituation sessions in this rat. These data are presented in Figures 5 and 6.

### Wavelet Analysis

The time–frequency characteristics of BP and HR functions were studied using a discrete wavelet analysis. This identified the contribution of particular bands of frequencies as a function of the time domain. The 0.025 Hz sGVS stimulus was confined to single bands of frequencies in the wavelet decompositions that were verified in previous studies (26, 33, 34). The signal was resampled so that its frequency was in the center of a band whose upper frequency limit was $\sqrt{2}*\mathrm{stim}\_\text{freq}$ and the lower frequency limit was $\mathrm{stim}\_\text{freq}/\sqrt{2}$. Because the vasovagal oscillation range was 0–0.2 Hz (34), only four low-frequency bands were analyzed in this study. Activity in Band 12, i.e., in an approximation band at 0–0.018 Hz, indicated that a transient response was induced, i.e., the low-frequency combined fall and return of BP and HR that constitute a vasovagal response (32). The other three bands were Band 11 (0.018–0.035 Hz), Band 10 (0.035–0.071 Hz), and Band 9 (0.071–0.141 Hz). Activity in Band 8 (0.141–0.282 Hz) and Band 7 (0.282–0.564 Hz) was minimal in previous experiments (26, 33, 34). In the experiments in this paper, the stimulus frequency was confined to Band 11, while adjacent bands (Bands 10 and 9) incorporated the second and fourth harmonics of stimulation, centered at twice and four times the stimulus frequency. The distribution of power in each frequency band comprising the signal was used as a metric for determining how the stimulus had generated the harmonics, and was used as a basis for comparing the response to different stimuli and to the same stimuli at different points in the experimental protocol. The power of each frequency band was computed as the average energy of the signal when it was reconstructed from frequency components in the band, calculated by (signal^2^/time). Each sample lasted 400 s and the original sampling interval was 16 ms to remove frequencies above 36.2 Hz, which were outside the range of interest. Using resampling and the db12 wavelet, the leakage from the band associated with the stimulus frequency to other bands was less than 5%, and a sinusoid at a single frequency had all of its energy in a single band of frequencies. The analysis was performed using Matlab (Mathworks, Inc., Natick, MA, USA). Standard deviations of wavelet-filtered responses for each frequency band were computed in order to compare results of wavelet decomposition of different data sets.

### Statistical Analysis

Changes in BP or HR were considered to be significant if they exceeded 2 SD of the pre-stimulus data. The dominant peaks at the frequency of stimulation and twice the frequency of stimulation were compared statistically using a Student’s paired *t*-test or a one way ANOVA with repeated measures applying a post Bonferroni adjustment. Changes in BP and HR were deemed significant when *p* < 0.05.

## Results

### Habituation from Repeated Testing (Unreported Data from Previous Experiments)

Our earlier studies employed single sinusoids, sGVS at different frequencies, ±70° oscillation in pitch, trains of sGVS at different frequencies from 0.2–0.25 Hz, 15–70° nose-up tilts, and translation while rotating (32). The results of these studies clearly indicated that ±2 and ±3 mA, 0.025 Hz sGVS and 70° nose-up tilts were potent stimuli for induction of vasovagal responses.

**Figure 1:**
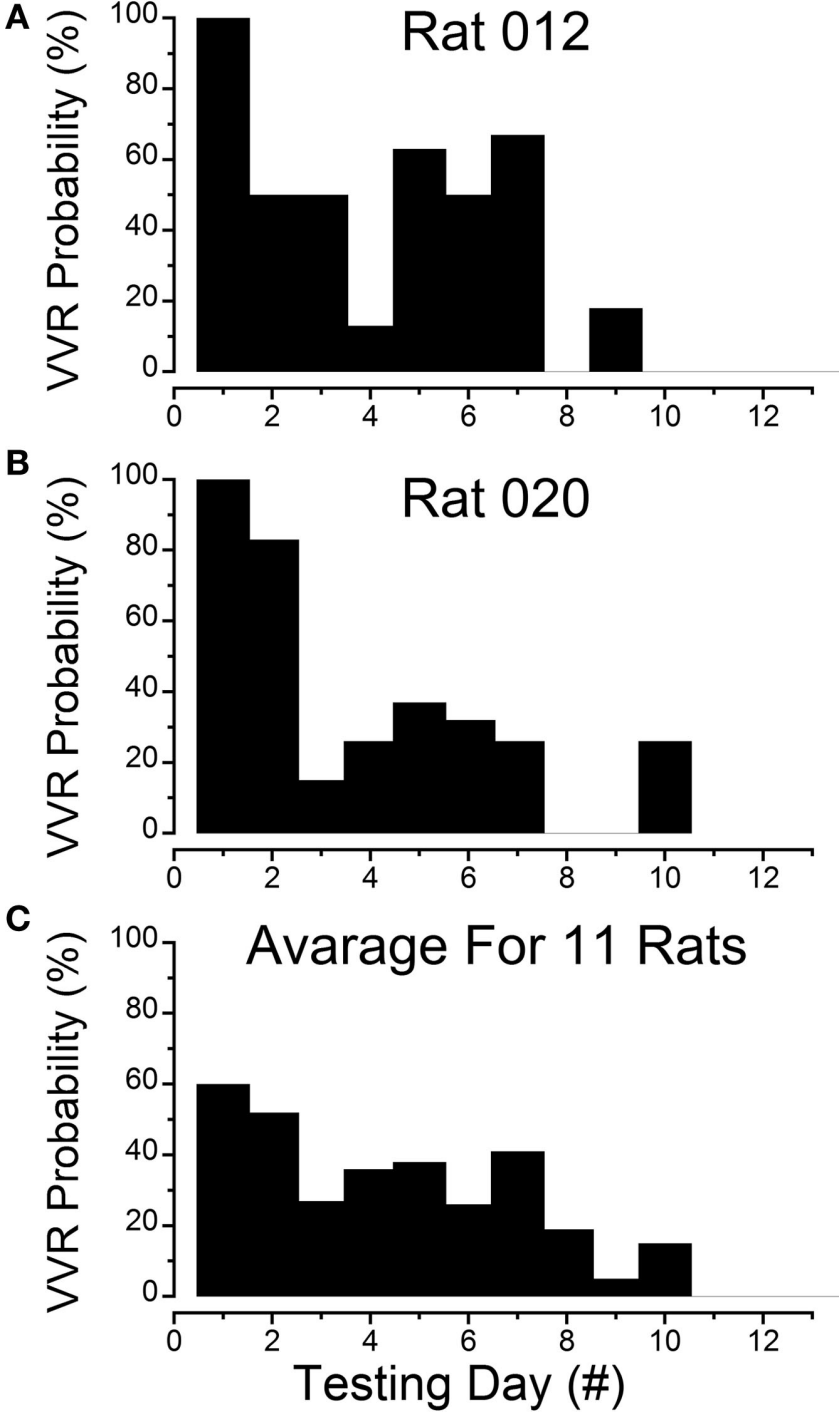
**Progressive loss of susceptibility to generation of vasovagal responses**. **(A)** Incidence of generation of vasovagal responses on repeated tests for R012 with ±3 mA, 0.025 Hz sinusoidal galvanic vestibular stimulation (sGVS) on the ordinate and sequential test days on the abscissa. The rat progressively lost its susceptibility until vasovagal responses were no longer induced by repeated testing on days 8, 10, 11, and 12. **(B)** Similar loss of susceptibility to generation of vasovagal responses for R020 induced by repeated stimulation with ±3 mA, 0.025 Hz sGVS. The incidence of vasovagal responses fell rapidly after day 2 and they could not be induced, despite repeated testing on days 8, 9, 11, and 12. **(C)** Loss of susceptibility in the 11 rats that form the basis of this report. Of the 11, 7 rats were repeatedly stimulated with the various forms of vestibular (otolith) stimulation over extended periods in previous studies. These seven rats lost their susceptibility progressively (see text for description). Also included are four rats (R009, R011, R012, and R020) that were habituated with ±2 and ±3 mA, 0.025 Hz sGVS over a 2-week period. The percentage of positive responses to the multiple test stimuli of the rats in the tested sample for each day of testing is shown on the ordinate and sequential test days on the abscissa. If the original group of rats did not respond to the multiple tests for 3 consecutive test days, they were removed from the sample after the third day. Despite the different time sequences in the original and later habituation procedures, the rats successively lost their susceptibility to the generation of vasovagal responses after approximately the same number of test sessions. As in **(A,B)**, the number of susceptible rats declined steadily until none were left by the 11th day.

The rats from these studies were selected for their initial susceptibility to the development of vasovagal responses using these stimuli and for their subsequent development of resistance to such vasovagal responses or vasovagal oscillations. Because the changes in behavior were maintained between stimulation sessions, despite irregular intervals between them, we considered the stimulus sessions to be sequential and disregarded the inter-stimulus intervals in order to compare stimulation results in the individual rats. The combined results from these seven rats are described in the text (below) and included in the results shown in Figure 1C.

Results of testing in seven rats and their progressive loss of susceptibility will be considered first.

These animals were repeatedly given the various forms of vestibular stimulation outlined above, and there was a continual loss of susceptibility to the induction of vasovagal responses as testing progressed. Tests included single and multiple sinusoids, pulses, oscillations in pitch, and tilts of various amplitudes. The most productive stimuli for inducing vasovagal responses were ±2 and ±3 mA, 0.025 Hz sGVS; 70° head-up tilt; or ±70° oscillation in pitch (26, 33, 34).

Testing of the animals occurred between 30 and 300 days. The number of tests varied from day to day and from animal to animal in these seven initial rats. To normalize the test results, the data were labeled as being from sequential test days, regardless of the time interval between them. The total number of tests for vasovagal responses each day was calculated, and the number of positive vasovagal responses was expressed as a percentage of the number of tests. The criterion for the loss of susceptibility was a failure to generate a vasovagal response on three consecutive days despite repeated testing. When this occurred, the animal was removed from the sample and no longer tested for induction of a vasovagal response. The total number of tests for vasovagal responses for each day and the total number of positive tests in which vasovagal responses were induced were registered. Based on these criteria, the frequency of occurrence for induction of vasovagal responses on each day was calculated, and plotted as a function of testing day. With the removal of unresponsive rats, seven animals were tested on days 1–4, six on days 5–7, five on day 8, two on days 9 and 10, and only one animal on day 11. Tests for vasovagal responses were performed approximately 30 times each day on days 1–7, except for day 6 when the animals were only tested 19 times. On days 8–11 the animals were tested 16, 9, 18, and 5 times, respectively. Vasovagal responses occurred during approximately 60% of tests on days 1 and 2, 40% on days 3–5, 21% on day 6, and 40% on days 7 and 8. Only one vasovagal response was induced out of 18 tests on day 10 (6%), and no vasovagal responses were induced on days 9 or 11. Thus, there was a continual loss of susceptibility to induction of vasovagal responses as testing progressed in these rats. Moreover, when a reduction in susceptibility occurred, it was maintained until the next test date, despite differences in the intervals between tests. This implies that changes in susceptibility were held for significant periods of time.

### Habituation with ±2 and ±3 mA sGVS

Four susceptible animals were used to determine the responses to intensive stimulation with sGVS, Rats R009, R011, R012, and R020. The sequence of loss of susceptibility in R012 and R020 are shown in Figures 1A,B. R012 had 15 tests each day and R020 had 10 tests each day, both with ±3 mA, 0.025 Hz sGVS. Initially, each rat had a vasovagal response to every ±3 mA, 0.025 Hz sGVS and/or every 70° nose-up tilt on the first day that they were tested. The response to these stimuli fell rapidly, and they became unresponsive by the third day of testing. Their responsiveness increased slightly over the next 3 days, but they were unresponsive on the seventh day. They responded to ≈10% of trials on the 8th day, and then became completely unresponsive and no longer responded from the 9th to 12th day of testing. Thus, there was a steady loss of susceptibility in these animals in response to repeated testing with sGVS and/or nose-up tilt.

The steady progression of the loss of susceptibility in R012, R020, R009, and R011 is plotted in Figures 1A,B, 3, 4A, 5, and 6A, and the cumulative loss of susceptibility in all 11 rats, disregarding intervals between training sessions, is plotted in Figure 1C. The steady decline in susceptibility was present in the whole group of 11 rats, despite the fact that the time between stimulations was different for the group of seven initial animals, and for the four rats that were more intensively stimulated. Every animal in the first group had at least one vasovagal oscillation or vasovagal response on the first day. The overall response on this day was 60%, and this responsiveness progressively fell so that there were no responders after day 10. The combined data confirm the loss of susceptibility in R009, R011, R012, and R020, but they have an additional implication. Since there were short and long periods between stimulations in the original group of seven rats, there must have been some storage of the habituation that had been attained by activation of the VSR in the original group. These data also indicate that habituation can be induced with shorter, intense periods of vestibular stimulation.

### Modification of BP and HR during Habituation

**Figure 2:**
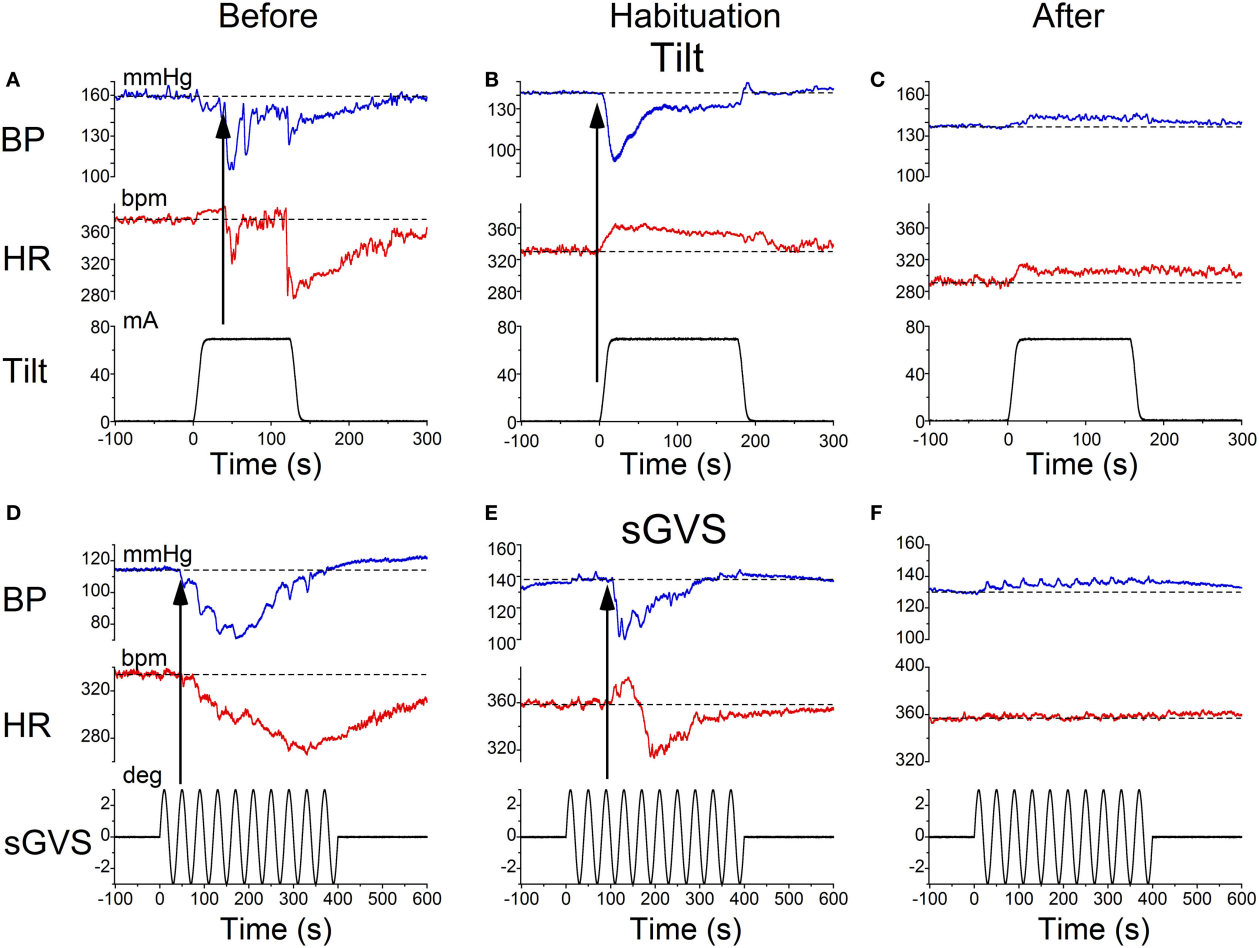
**Changes in blood pressure (BP) and heart rate (HR) during habituation in R009**. **(A–C)** Changes in BP (top traces) and HR (middle traces) in response to 70° nose-up tilt (bottom traces) at three stages during habituation. **(A)** Initially, both BP and HR fell in response to the 70° nose-up tilt. **(B)** As habituation progressed, the tilt induced a fall in BP, but a rise in HR of ≈45–50 bpm that persisted for ≈250 s. **(C)** At the end of habituation, the nose-up tilt now induced only small increases in both BP and HR. **(D–F)** Changes in BP (top traces) and HR (middle traces) in response to stimulation with ±2 mA, 0.025 Hz sGVS (bottom traces). **(D)** Initially, both BP and HR fell together. The fall in HR was slower and persisted for over 10 min. The maximum fall in HR was ≈50 bpm. **(E)** Later, during habituation, the fall in BP was initially countered by a ≈25 bpm increase in HR that lasted for ≈100 s before HR also fell. **(F)** Finally, there was only a small rise in both BP and HR in response to the sGVS. BP increased by ≈10 mmHg and persisted for over 10 min. The rise in HR was also small and lasted for more than 10 min. The data for the tilt and sGVS were taken on the same days during the initial tests **(A,D)**, during the middle stages **(B,E)**, and at the end of the habituation process **(C,F)**. In comparison, the changes produced in the intermediate sessions **(B,E)** were more profound with the ±2 mA sGVS than with the 70° (0.91 *g*) nose-up tilt.

Characteristic changes in BP and HR were produced by habituation in each of these rats. Here, two examples are shown. In the first, before conditioning, R009 developed a vasovagal response on each of nine tests. About 50 s after a 70° nose-up tilt, there was a drop in BP of ≈50 mmHg followed by a reduction in HR of ≈35 bpm (Figure 2A). The animal developed a second drop in BP and HR when it was tilted back to the nose-up position, demonstrating its susceptibility to development of vasovagal responses. In this instance, the decline in HR only slightly outlasted the decline in BP. On the same day, activation with ±2 mA, 0.025 Hz sGVS (Figure 2D) also induced typical vasovagal responses ≈45 s after the onset of the stimulus (Upward arrow). There was a drop in BP of ≈50 mmHg and in HR of ≈50 bpm. The decline in BP lasted ≈300 s, and the decline in HR continued past the end of the trace beyond 600 s (10 min). During the course of habituation, the 70° nose-up tilt again caused a ≈35 mmHg drop in BP (Figure 2B), but now this was countered by an increase in HR (from 285 to 295 bpm) that lasted beyond 300 s (Figure 2B). The ±2 mA, 0.025 Hz sGVS appeared to be somewhat stronger in generating a vasovagal response and it produced a drop in BP of ≈50 mmHg, and a drop in HR of ≈50 bpm (Figure 2E). Now, however, there was an initial increase in HR of ≈30 bpm. The HR rose to ≈360 bpm followed by a ≈40 bpm decline to ≈320 bpm, which lasted ≈1 min (Figure 2E). The vasovagal response in this instance was similar to the Phase 2 response in the development of vasovagal responses described by Julu et al. in humans; i.e., in which HR initially countered the drop in BP but was only partially successful (7). Finally, at the end of the habituation process, both the 70° nose-up tilt (Figure 2C) and the ±2 mA, 0.025 Hz sGVS (Figure 2F) only produced small increases in BP and HR that lasted for the duration of the stimulus.

**Figure 3:**
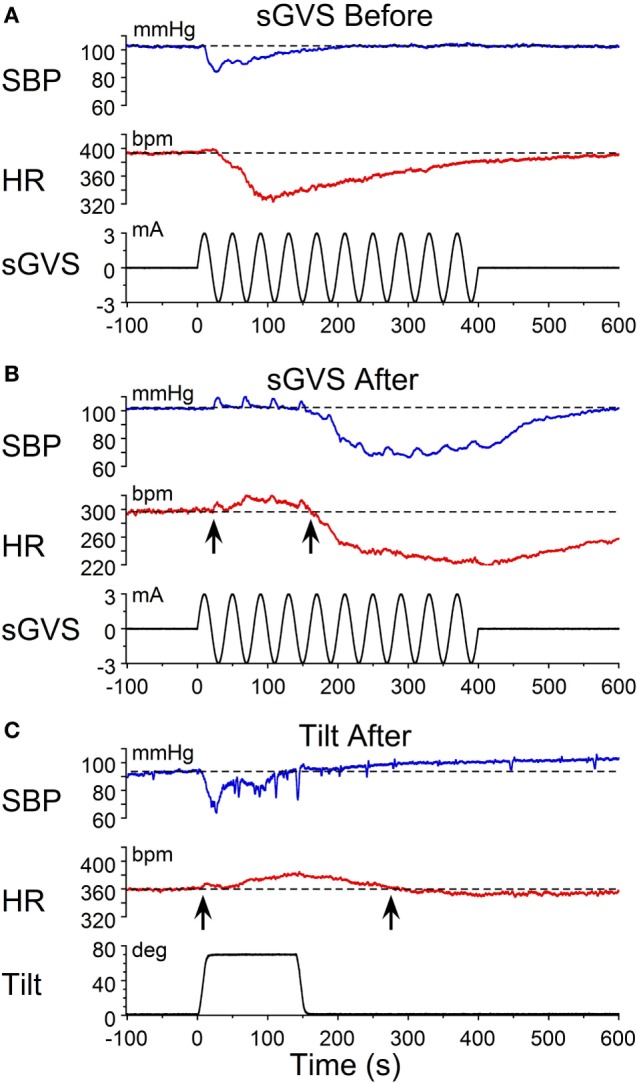
**Changes in blood pressure (BP) and heart rate (HR) during habituation in R011**. Changes in BP (top traces) and HR (middle traces) in response to ±3 mA, 0.025 Hz sGVS (bottom traces). **(A)** There was an initial fall in both BP and HR that was larger and lasted longer for the HR. **(B)** During the habituation process, initially, there were several, small transient increases in BP before BP fell in response to the sGVS. There was also a ≈20 bpm initial increase in HR (first upwards arrow) that persisted for ≈150 s before HR also fell (second upwards arrow). **(C)** At the same time as in B, nose-up tilt caused a drop in BP, but a concomitant rise in HR of ≈30 bpm (first upwards arrow) that persisted for nearly 300 s (second upwards arrow). The difference in the response of the HR to ± 3 mA sGVS and the 70° nose-up tilt suggests that the sGVS was a more powerful stimulus to the otolith system than the 70° nose-up tilt.

Similar results were obtained in R011, the susceptible rat from this group stimulated with ±3 mA, 0.025 Hz sGVS. Initially, the sGVS caused a drop in BP of ≈20 mmHg and a fall in HR of ≈100 bpm that outlasted the drop in BP (Figure 3A). During habituation, the same stimulus caused a more profound drop in BP. This was partially countered by an initial ≈50 bpm rise in HR over the first ≈100 s, before HR fell for more than 600 s (10 min) (Figure 3B). A similar rise in HR was induced that countered a brief fall in BP produced by a 70° nose-up tilt at the same time in the habituation process in this rat (Figure 3C). Thus, there was a consistent increase in HR during habituation that countered the falls in BP, until there was only a small rise in both BP and HR in response to the vestibular stimuli (Figure 3C). These examples demonstrate that the habituation was induced in both of these rats by an activation of the heart, which opposed the falls in BP. Such activation of HR strongly suggests an increase in baroreflex sensitivity.

### Progressive Changes in Susceptibility and HR during Habituation

**Figure 4:**
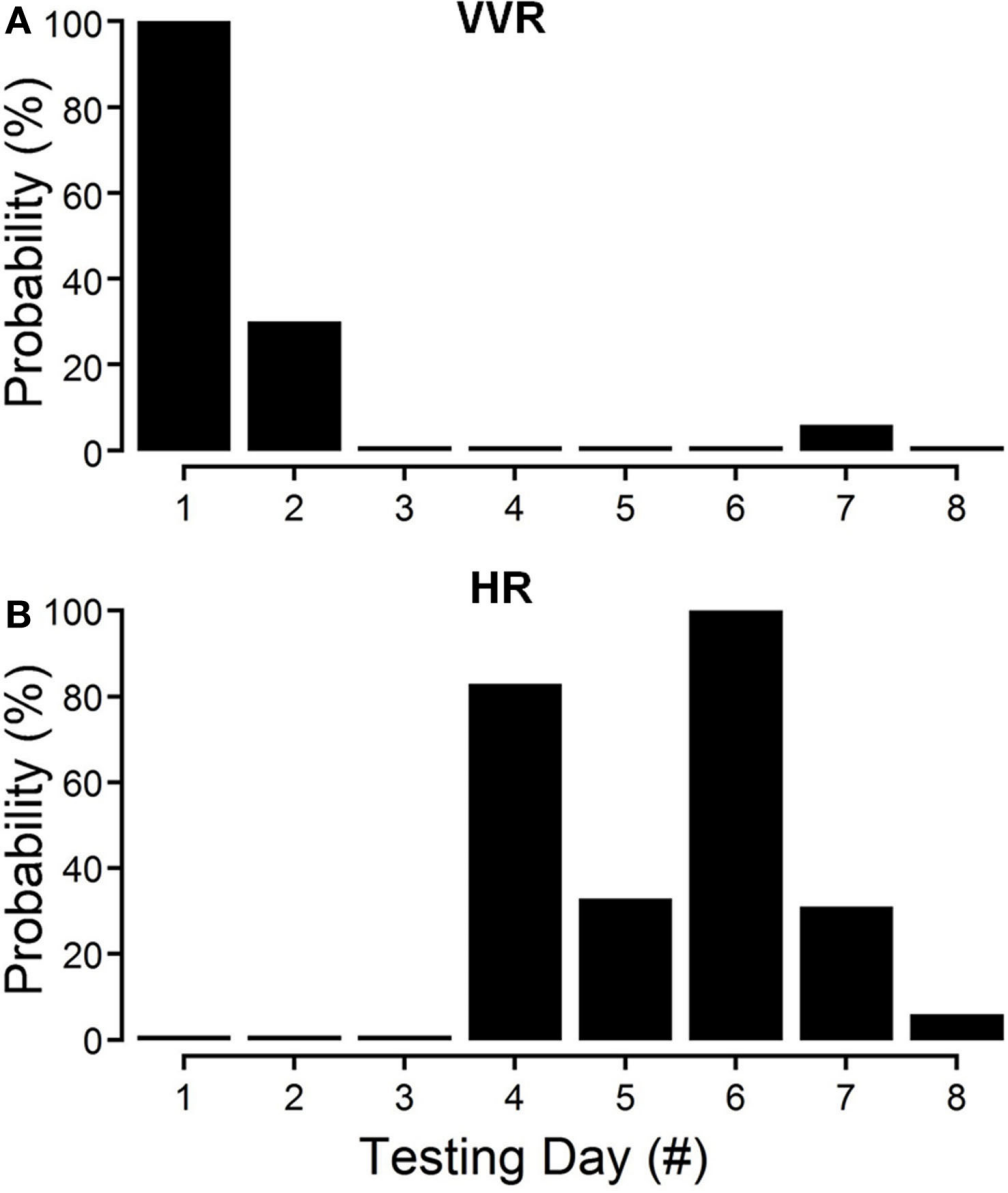
**Progressive decline in susceptibility to generation of vasovagal responses and coincident increase in heart rate (HR) during habituation in R009**. The days of habituation are represented sequentially on the abscissae (see Materials and Methods). **(A)** Susceptibility fell rapidly over the first 2 days. **(B)** HR increased dramatically on the fourth day of testing, peaked on the sixth test day, and then fell rapidly over the next 2 test days. Finally, there was only a minimal response in blood pressure and HR by the eighth day, when the animal had become habituated. These data demonstrate that the fall in susceptibility was associated with a transient increase in HR that then fell back as the habituation proceeded.

The changes in susceptibility and HR in R009 demonstrate the progressive fall in susceptibility and the rise in HR as a function of the habituation process. The animal was habituated with ±2 mA, 0.025 Hz sGVS. The ±2 mA sGVS was used to determine whether this stimulus, which has been widely used in studies of MSNA, would also produce habituation. Initially, R009 had a vasovagal response every time it was tested (Figure 4A, day 1), but it shortly developed resistance, and only responded to ≈30% of the tests on day 2. Beginning on day 4 (Figure 4B), there was a dramatic 80% rise in HR that was also present on day 6, and then fell to 10% above resting level by day 10. Concurrently, the sGVS only caused a minimal decrease in BP. These data and those in Figures 2–4 also raise the possibility that it was activation of the heart through the baroreflex, in response to the fall in BP and HR that countered the drop in BP and brought the rat back to normality.

### Temporal Progression of Habituation

**Figure 5:**
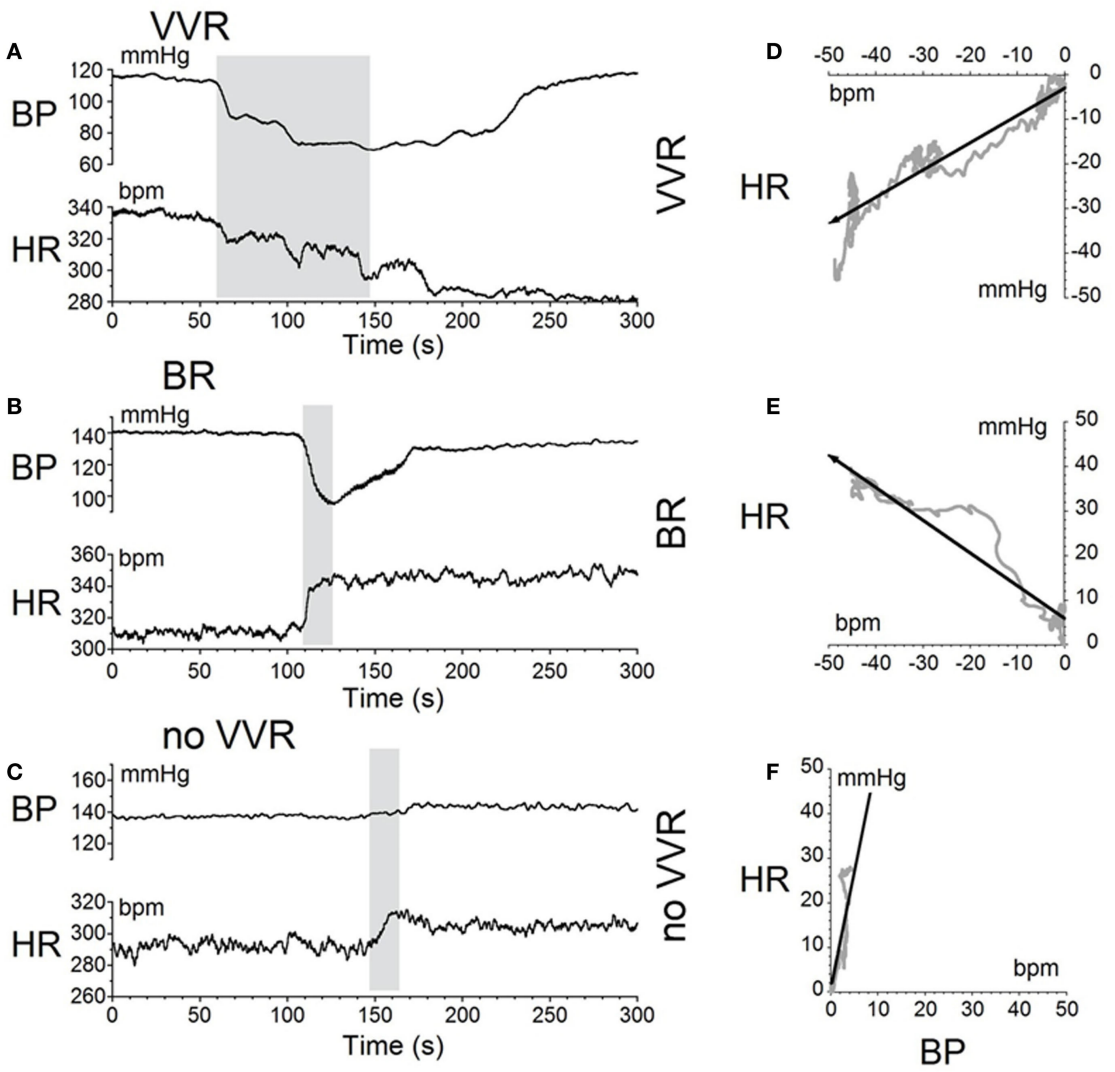
**Sequential changes in blood pressure (BP) and heart rate (HR) during habituation in R009**. **(A–C)** Graphs of changes in BP (top traces) and HR (bottom traces) induced by activation of the vestibulosympathetic reflex with 400 ms trains of ±2 mA, 0.025 Hz sGVS at the beginning, during, and at the end of habituation. The trains of sGVS caused different slopes of the combined responses in BP and HR at the different times during the habituation process. **(A)** At the beginning of habituation, both BP and HR fell together. The decline in HR persisted for more than 100 s. **(B)** During habituation, BP fell, but there was an increase in HR that persisted for >100 s. **(C)** At the end of habituation, BP was essentially unaffected, but there was a small rise in HR. **(D–F)** The slopes of the combined changes in BP and HR from **(A–C)** were derived from a least squares regression of these recordings. The vectors were originally in the 180–240° quadrant **(D)**, then in the 240–360° quadrant during habituation **(E)**, and finally in the 0–90° quadrant after habituation had taken place **(F)**.

**Figure 6:**
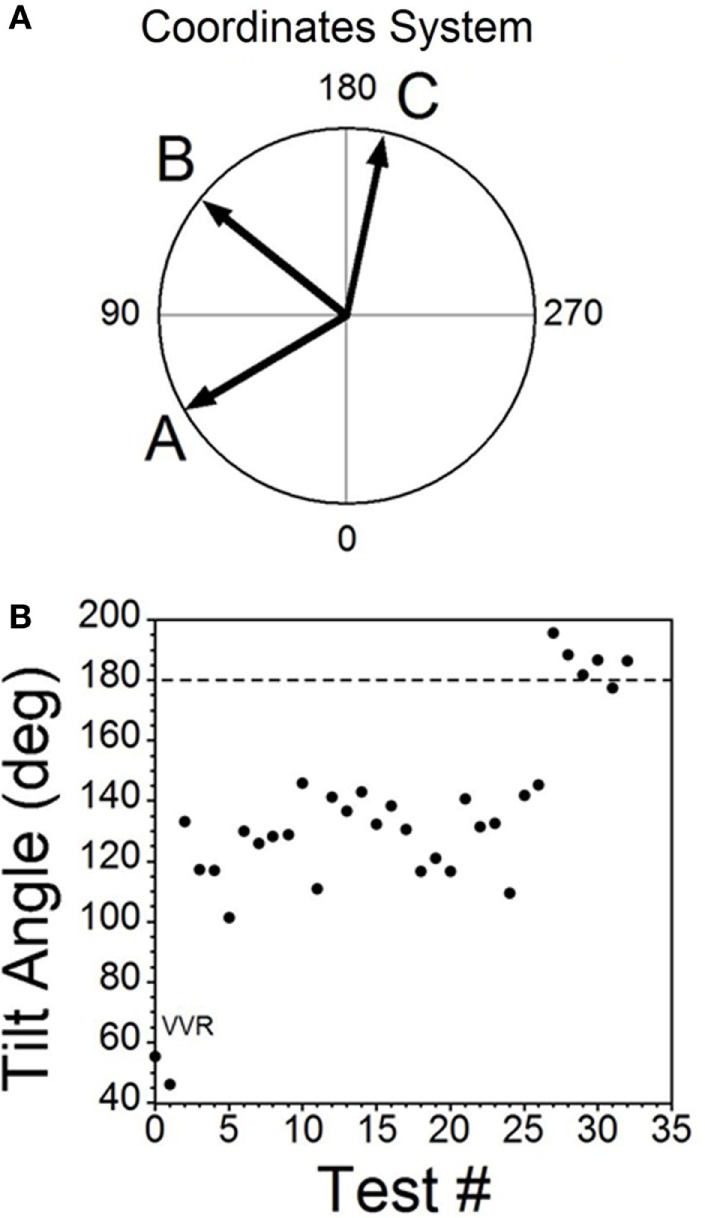
**Consecutive changes in blood pressure (BP) and heart rate (HR) during habituation in R009**. **(A)** Plots of the vectors from Figures 5D–F are given in polar coordinates. This graph demonstrates the progressive changes in BP and HR during habituation. **(B)** Changes in the slopes of the BP/HR relationship as R009 became habituated plotted from the first to the last tests (bottom to top). The angle of tilt of the vector formed from BP and HR is shown on the ordinate, and the sequential test numbers are shown on the abscissa. There were 30 tests during the habituation procedure and 3 tests after the animal was habituated. The relationship between BP and HR systematically and gradually changed during habituation. There was a continual increase in the angle of the vector formed from BP and HR. The dashed line at 180° was achieved by the 27th test and was maintained through the 33rd test. Thus, there was a steady change in BP and HR from the beginning to the end of habituation.

The moment-to-moment changes in BP and HR were characterized further during the various phases of habituation in R009 to demonstrate the continuous progression of BP and HR more completely as R009 became habituated. The changes in BP and HR during habituation were initially characterized at three stages in the process, using the coincidence that the changes in both parameters were of similar magnitudes. That is, both BP and HR fell by ≈20–50 mmHg or ≈20–50 bpm at the onset of a vasovagal response and then slowly recovered toward their original values. The recovery of the responses were fitted with a least square linear regression starting from the largest changes in BP and HR, and extending toward the origin, which contained the original values of BP and HR before stimulation (Figures 5A,C). The direction of the recovery toward the pre-stimulus values is shown by the adjacent arrows (Figures 5D,F). These vectors are shown in a polar representation (Figure 6A). Initially, the direction of recovery toward the origin (0°) was similar in both BP and HR, i.e., in the 180–240° quadrant (Figures 5B and 6A), Later during the habituation process, HR opposed the drop in BP. Although BP started from a depressed level, the fall in BP was opposed by an increase in HR (Figure 5D), so that the combined curve fell toward the origin in the 240–360° quadrant (Figure 6A). Later, both BP and HR rose and then fell together toward their original values in the 0–90° quadrant (Figures 5F and 6A).

Similar plots were made for BP and HR in each of the 30 tests during the habituation of R009 and for three tests made on the day after the end of habituation (Figure 6B). They demonstrate a continuous modification of the relationship between BP and HR over the process of habituation. The values progressed at an increased tilt angle until they finally reached the values shown by the tilt angles in Figure 6B. Thus, the simultaneous drops in BP and HR had been continuously modified by the ±2 mA, 0.025 Hz sGVS so that increases in HR initially countered subsequent drops in BP for several trials, after which HR rose above its resting level to counter the drop in BP. Finally, both BP and HR became much less responsive to further stimulation, and this condition was maintained for three additional tests given over the next several weeks.

### Activation of the VSR

**Figure 7:**
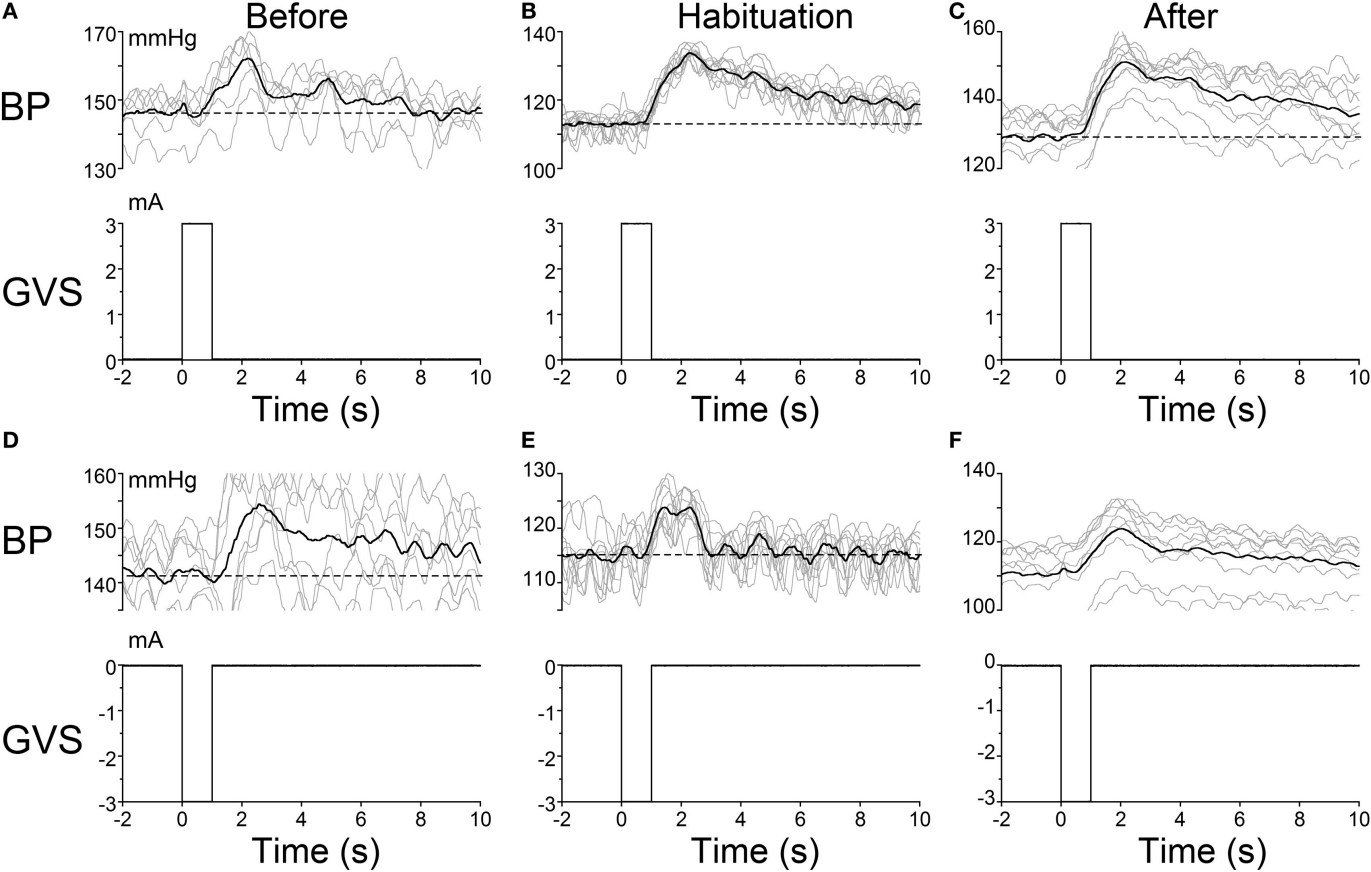
**Pulse stimulation of the vestibulosympathetic reflex before, during, and after habituation in R012**. Increases in blood pressure (BP) (top traces) from +3 mA **(A–C)** and −3 mA **(D–F)** 1 s pulses of Galvanic Vestibular Stimulation. The stimuli induced increases in BP of ≈10–25 mmHg and were somewhat larger when the initial BP was lower. The BP rose rapidly at the onset of stimulation before, during and after habituation. These data demonstrate that the response of the cardiovascular system to vestibular stimulation was not significantly affected by the habituation process. There were no changes in HR (not shown).

The habituating stimulus, sGVS, primarily activates the vestibular (otolith) system and the VSR. We questioned whether the repetitive stimuli to the vestibular nerves induced by sGVS had made the VSR unresponsive. Further experiments demonstrated that this was not the case. Normally, current pulses and sinusoids of sGVS induce increases in BP, similar to the increases that occur in humans in changes of posture relative to gravity (orthostasis) (29), and HR is not generally activated by these stimuli unless the stimulus currents are large (34). To study whether the responsiveness of the vestibular nerve had been altered by the habituation process, a rat that was habituated with ±3 mA, 0.025 Hz sGVS (R012, Figure 1A), was given 1 s ±3 mA pulses of sGVS before, during, and after habituation (Figure 7).

Activation of the VSR with + 3 mA sGVS continued to induce increases in BP before (≈150–162 mmHg lasting for 8 s), during (≈120–132 mmHg lasting for 10 s), and after habituation (≈130–152 mmHg lasting for 10 s; Figures 7A–C). Results were similar for −3 mA pulses of sGVS before (≈140–155 mmHg lasting for 10 s), during (≈115–123 mmHg lasting for 3 s), and after habituation (≈110–125 mmHg lasting for 10 s; Figures 7D–F). There were no changes in HR with these stimuli. Thus, habituation had not altered the response of the VSR to vestibular (otolith) stimulation, although such stimulation no longer induced vasovagal oscillations or vasovagal responses.

### Low-Frequency Activity in BP and HR

In previous wavelet analyses, we demonstrated that vasovagal oscillations and vasovagal responses could only be induced when there was significant 0.025 and 0.05 Hz low-frequency activity in both BP and HR, and that this activity disappeared when rats were no longer susceptible to the generation of vasovagal oscillations or vasovagal responses (33, 34). Such low-frequency activity has also been demonstrated in a human fainter associated with a faint (36) and in alert dogs after withdrawal of blood, presumably when the dogs were in a pre-syncopal state (43, 44). We questioned whether there would be increased activity in the Bands that contain the 0.025 and 0.05 Hz oscillations in BP and HR when the rats were susceptible to the generation of vasovagal responses and during the active phases of habituation, and we predicted that these low-frequency oscillations would disappear as a consequence of the habituation.

**Figure 8:**
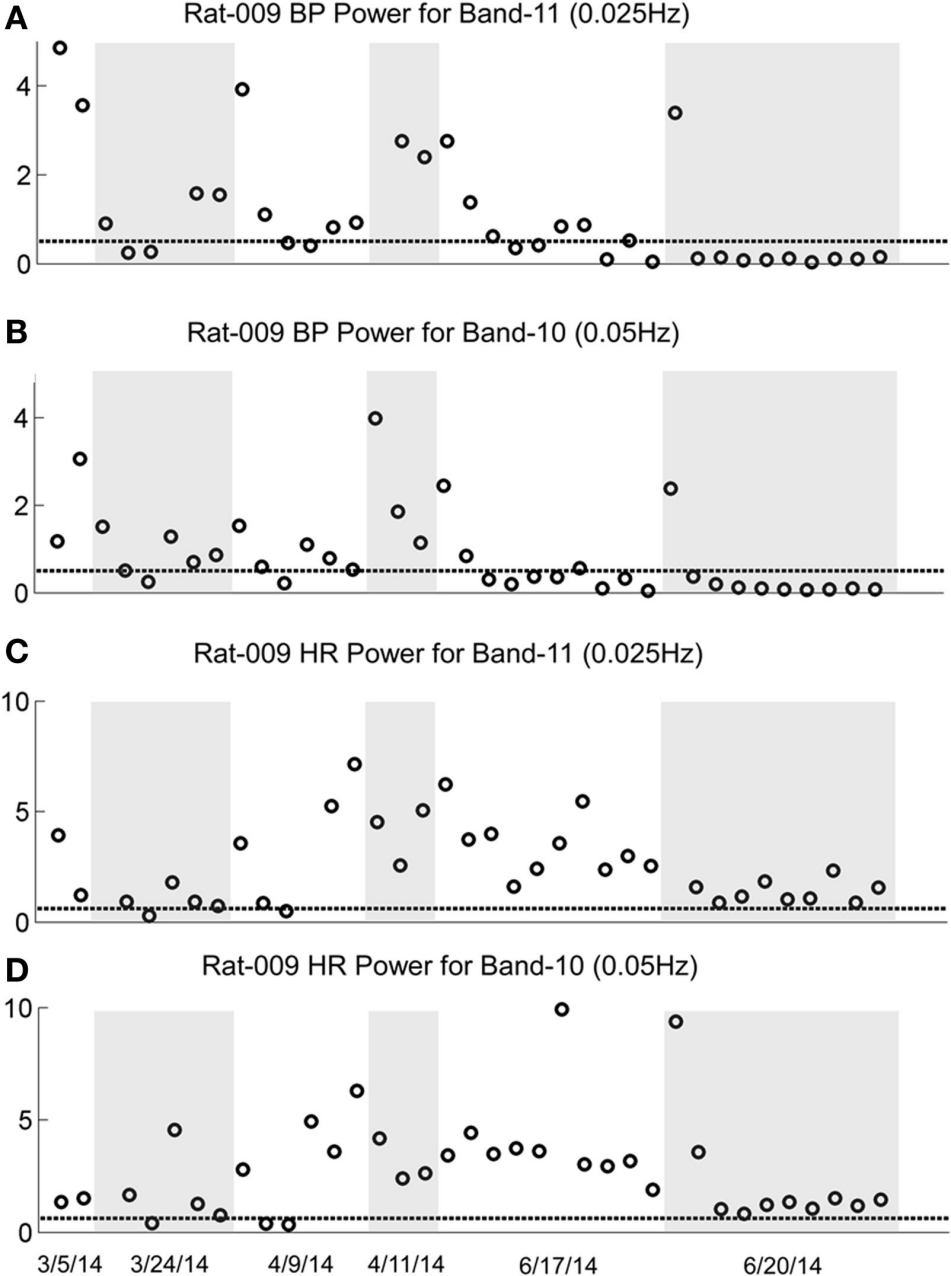
**Changes in low-frequency oscillations of blood pressure (BP) and heart rate (HR) during habituation in R009**. The four traces show the low-frequency oscillations in BP in Power Bands 11 (0.025 Hz; **A**), and 10 (0.05 Hz; **B**), and HR for Power Bands 11 (0.025 Hz, **C**), and 10 (0.05 Hz, **D**) before, during, and after habituation. The circles above the abscissa reflect the power of the low-frequency components in the BP and HR signals for the duration of the stimulation, and the dashed, horizontal line reflects the upper limit of oscillation in the normal rat when no stimulation was applied. The ordinate reflects the power (energy), which is calculated by computing the total energy of a particular band, divided by the length of the signal. The six dates below the bottom trace indicate some of the dates on which the data were taken. The circles on each graph indicate the sequential changes in BP and HR over the period of testing and habituation. The initial testing to determine that the rat was susceptible to the generation of vasovagal responses took ≈10 weeks. The actual habituation in R009 occurred over 2 weeks from 6/6/14 to 6/20/14. The low-frequency oscillations disappeared promptly after the onset of habituation and remained at or close to normal levels for both bands 11 and 10 in BP and HR. The increases in HR in Bands 11 and 10 came close to the normal levels upon habituation, but did not fall below it. There were no significant low-frequency oscillations in HR toward the end of habituation. These data demonstrate a general loss of low-frequency oscillations when R009 had reached habituation.

The changes in Band 11 (0.025 Hz) and Band 10 (0.05 Hz) in BP and HR in R009 during a several month pretest period (3/5/14–6/4/14) and the condensed 2-week period of habituation, are shown in Figure 8. The horizontal dashed lines in the BP and HR traces indicate the upper limit of the low-frequency oscillations in Bands 11 and 10 for both BP and HR, and were determined using rats that had not been stimulated. The presence and amplitude of these frequencies are shown on the ordinate, with each circle representing the result of a separate determination, listed sequentially. However, the intervals between individual tests varied according to the demands of the experiment. There was a substantial amount of low-frequency activity present in both BP and HR until shortly after the habituation procedure began.

Then, both low frequencies of BP and HR fell toward normal levels. The fall was more substantial in BP than in HR but even HR was maintained close to normal levels. Thus, there was substantial low-frequency activity in both BP and HR during the prolonged pretest period that promptly disappeared after the onset of the vestibular stimulation at the time when vasovagal responses could no longer be induced in this rat.

**Figure 9:**
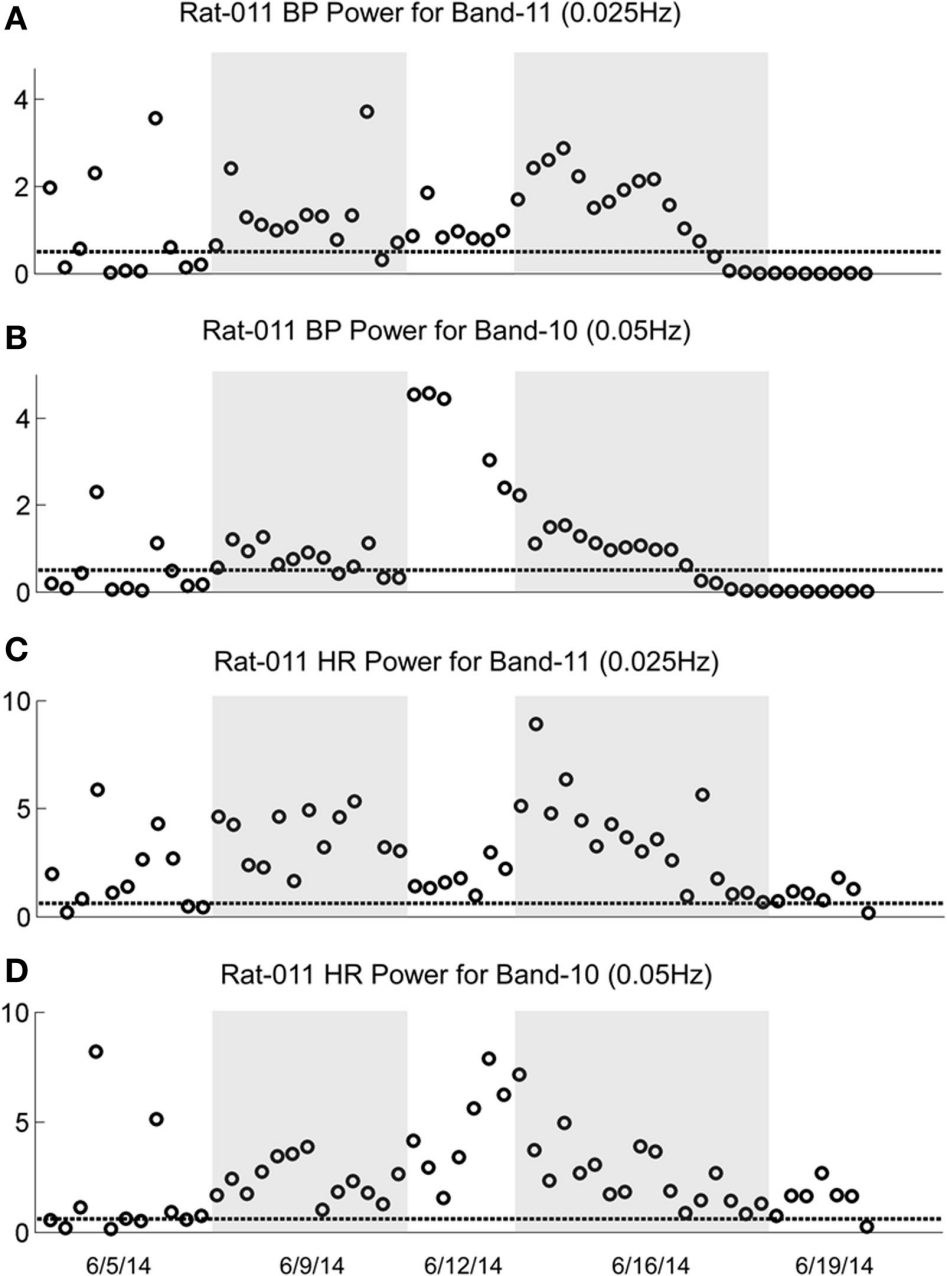
**Changes in oscillation of blood pressure (BP) and heart rate (HR) during habituation in R011**. The four traces show the low-frequency oscillations in BP for Power Bands 11 (0.025 Hz, **A**) and 10 (0.05 Hz, **B**) and HR for Power Bands 11 (0.025 Hz, **C**) and 10 (0.05 Hz, **D**) before, during, and after habituation. Habituation began after initial testing to determine that the rat was susceptible to generation of vasovagal responses. In this segment, the total time of intense habituation occurred over 2 weeks from 6/5/14 to 6/19/14. The five dates below the bottom trace indicate some of the dates on which the data were taken. The circles above the abscissa reflect the power of the low-frequency components in the BP and HR signals for the duration of the stimulation, and the dashed, horizontal line reflects the upper limit of oscillation in the normal rat when no stimulation was applied. The ordinate reflects the power (energy), which is calculated by computing the total energy of a particular band, divided by the length of the signal. The circles on each graph indicate the sequential changes in BP and HR over the period of testing and habituation. Initially, there were significant increases in the low-frequency oscillations in both BP and HR in Bands 11 and 10 that finally fell to normal or close to normal levels in the last nine tests. The increases in BP fell below the normal level for bands 11 and 10, toward the end of the habituation. The increases in HR in Bands 11 and 10 came close to the normal levels upon habituation, but did not fall below it. There were no significant low-frequency oscillations in HR toward the end of habituation. These data demonstrate a general loss of low-frequency oscillations when R011 had reached the habituated state.

The sequence of the changes in BP and HR produced by the habituation procedure in R011 from 6/6/14 to 6/20/14 was similar to those in R009, but the pretest procedures were done rapidly, over a several day period, and it took longer for the low-frequency activity (0.025 and 0.05Hz) in both BP and HR to fall below or close to normal levels (Figure 9). Nevertheless, it was not possible to induce vasovagal responses or vasovagal oscillations in either rat by the end of habituation as shown in Figures 2C,F and 3C. Therefore, we can conclude that habituation had been successful in reducing the susceptibility to generation of vasovagal responses by blocking occurrence of low-frequency oscillations in BP and HR in these rats.

### Baroreflex Sensitivity

**Figure 10:**
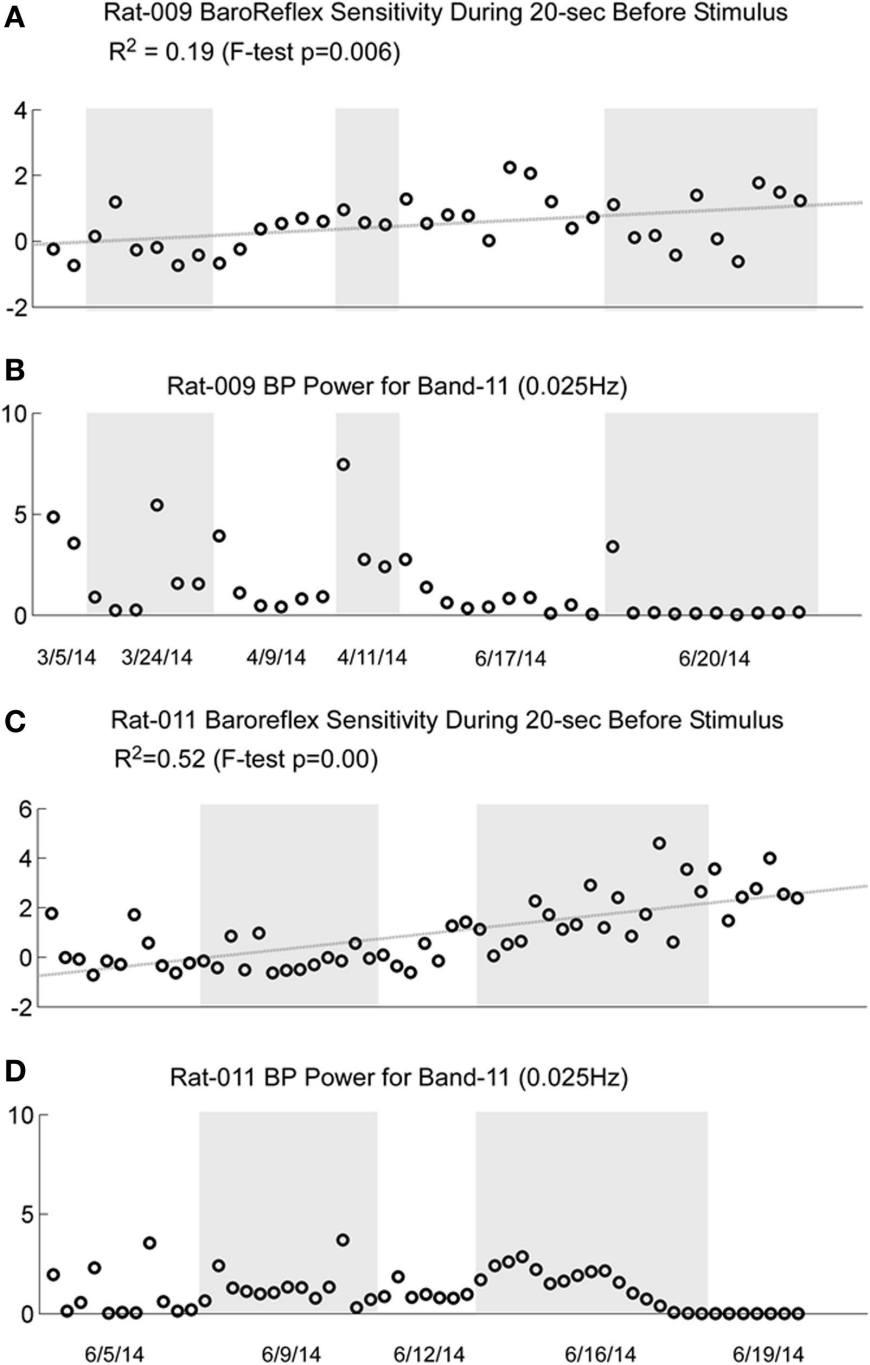
**Comparison of the increase in baroreflex sensitivity and blood pressure (BP) Power Band 11 responses (0.025 Hz) for R009 and R011**. The four traces show the increase in baroreflex sensitivity (**A** and **C**) and low-frequency oscillations in BP for Power Band 11 (**B** and **D**) for R009 and R011. The increases in baroreflex sensitivity were significant in both animals (R009: *p* = 0.006, R011: *p* < 0.001). The *R*^2^ value was higher in R011 than in R009, indicating that there was a higher linear correlation for R011. There was no dramatic change in baroreflex sensitivity that was associated with the loss of low-frequency oscillations in BP and HR in both animals. The traces of the low-frequency oscillations in BP were repeated to demonstrate that changes in these oscillations were not reflected specifically in the changes in baroreflex sensitivity. Regardless, these data demonstrate that baroreflex sensitivity could significantly increase as a result of the vestibular stimulation and the habituation process.

There is a reduction in baroreflex sensitivity in the anesthetized state (45–48). Presumably, this reduction contributed to the loss of susceptibility of the generation of vasovagal responses. As shown in Figures 2A,D and 3A, both BP and HR fell together, and the baroreflex did not counter the combined fall in BP and HR until habituation began (Figures 2B,E and 3B). We questioned whether baroreflex sensitivity was altered during the progressive habituation induced by the consistent use of sGVS. As shown in Figure 10 (first and third traces), there was a steady increase in baroreflex sensitivity in the two susceptible rats that lost their susceptibility during the habituation process (*p* < 0.01 for both rats). Each circle in Figures 9A,D represents a separate determination, listed sequentially. Although sequential, the intervals between individual tests varied according to the demands of the experiment. The increase in baroreflex sensitivity began during the preliminary testing, in R009, and continued through the habituation period in both rats. Traces of the low-frequency (0.025 Hz) activity are repeated to demonstrate that there was no inflection in the rise in baroreflex sensitivity during habituation (second and fourth traces). This is particularly notable for R011, which was habituated intensively from its susceptibility state.

These data are compatible with the postulate that an increase in baroreflex sensitivity plays a role in the habituation of vasovagal responses, and there was no similar increase in baroreflex sensitivity in several rats that failed to become habituated with ±70° oscillation in pitch (not shown). Nevertheless, these data imply that an increase in baroreflex sensitivity was not solely responsible for the reduction in susceptibility to the generation of vasovagal responses. That is, the sequence of changes in baroreflex sensitivity is not in close accord with the sequence of development of habituation. Baroreflex sensitivity increased steadily in R009, but it was possible to induce vasovagal responses at the onset of habituation (Figures 2A,D) almost three months after testing had begun.

## Discussion

This study shows that the susceptibility of anesthetized rats to the induction of vasovagal responses can be effectively reversed by activation of the VSR. This was demonstrated in two sets of rats: seven rats that were initially susceptible to the generation of vasovagal responses and were utilized in other experiments lost their susceptibility when they were stimulated at different intervals with various forms of vestibular stimulation (Figure 1C). These studies were directed to other ends, and it was not possible to isolate the specific stimuli that had caused the loss of susceptibility to the generation of vasovagal responses nor to determine the specific changes in BP and HR that accompanied the habituation process. Thus, the prime goal of the present study was to uncover the actual changes in BP and HR that had been responsible for the habituation. This was shown by the close study of four rats that were susceptible to the generation of vasovagal responses and lost that susceptibility when the VSR was repeatedly stimulated with ±2 and ±3 mA, 0.025 Hz sGVS. The loss of susceptibility was associated with an initial and then a more substantial rise in HR to oppose the fall in BP. Finally, the same stimuli only caused a slight increase in BP and HR. After habituation, vasovagal responses could no longer be induced by any of the vestibular (otolith) stimuli including 70° nose-up tilt and ±3 mA sGVS. These findings were supported by the loss of low-frequency oscillations (0.025 and 0.05 Hz) in BP and HR (Figures 8 and 9) that have been shown to be essential for activation of vasovagal oscillations and vasovagal responses in anesthetized rats, dogs, and humans (33, 34, 36, 43, 44). The habituation was also associated with a significant rise in baroreflex sensitivity (Figure 10). To our knowledge, this is the first demonstration of the processes that are likely to underlie the habituation of syncope, as well as the changes in HR and BP that resulted in the loss of susceptibility to the induction of vasovagal oscillations and vasovagal responses. Here, we consider the characteristics of BP and HR that were produced by repeated activation of the VSR, speculate on the central mechanisms that could have been involved in producing the loss of susceptibility, and propose potential clinical implications of these findings.

Development of habituation took a characteristic, continuous course, shown by the modifications in BP and HR that occurred in each of the rats during habituation. These changes were demonstrated in a typical rat (R009), using the coincidence of similar numerical values for BP and HR at the onset of habituation to compare the relative changes in each parameter. This comparison provided insight into the nature of the process that alters BP and HR during habituation, namely, a significant but limited rise in HR rather than the combined fall in BP and HR (Figure 4) that are the essential components of the vasovagal response. Thus, although the rats were under anesthesia where baroreflex sensitivity is low (49–51), a significant increase in baroreflex sensitivity was associated with the habituation during which the rat cardiovascular system became impervious to strong activation of the VSR. However, the slope of the restoration in baroreflex function consistently increased as habituation proceeded, and during the time it took to determine if R009 was suitable, there was a continuous rise in baroreflex sensitivity at a time when it was still possible to drive the rat into vasovagal responses. This is shown quite clearly in Figures 2–4 that demonstrate that both of the rats were susceptible to the generation of vasovagal responses on 6/5/14 and 6/6/14 when the habituation procedures began, and when vasovagal responses could be induced (Figures 2A,D and 3A). A comparison of the rise in baroreflex sensitivity, in both rats (Figure 10, first and third traces) demonstrates that the rise in sensitivity was smooth and showed no inflection on or about 6/5/14 and 6/6/14 when there was a dramatic turnaround in the low-frequency oscillations (Figure 10, second and fourth traces) that were associated with the loss of the ability to generate vasovagal responses. Thus, although baroreflex sensitivity may have a critical role in reactivating HR to oppose drops in BP, events around the production of low-frequency oscillations (i.e., 0.025 and 0.05 Hz) are likely to be critical elements in initiating vasovagal responses and vasovagal syncope (33, 34, 36), and their disappearance is a critical part of the habituation process.

Also lacking from this formulation are the specific trigger for the combined drop in BP and HR during the vasovagal responses as well as the source of the signal that reverses the reduction in function of the baroreflex. A model showing how the baroreflex and VSR impinge on the cardiovascular system suggests that there is a critical variable that governs the onset of the vasovagal response (14), namely an element labeled desired BP (Desired BP), which is comprised of the diastolic/systolic amplitudes. When the amplitude of the Desired BP fell in the simulations, there was an initiation of a reduction in both BP and HR, leading to a simulated vasovagal response by the model. Where such a signal arises is unknown, but it could come from the interaction of the uvula with the VSR and the cardiovascular system (52). Direct projections extend from the uvula to the otolithic portions of the Vestibular Nuclei to the parabrachial nuclei and the Nucleus Tractus Solitarius (NTS) that receive the input from the baroreflex receptors (53–57). There is also a disynaptic projection from the uvula to NTS that could control baroreflex sensitivity (29, 52, 58–61). If this is correct, then projections from the uvula could be the critical input to control susceptibility to sympathetic activation of syncope, regardless of whether it arises from direct activation of the VSR or from one of the many other causes of vasovagal syncope that project through the parabrachial nuclei. It is also possible that among the inhibitory processes generated onto the baroreflex is a clamp on the low-frequency activity from inhibitory input that arises in the uvula. If this speculation is correct, then the increased low-frequency activity in BP and HR could be a reflection of cerebellar-generated activity that controls baroreflex sensitivity that inhibits production of syncope and promotes generation of MSNA (62).

### Clinical Implications

Our findings support the studies of Ector, Kinay, Reybrouck, and Di Girolamo (17, 18, 20, 21, 63), which habituated vasovagal syncope with static head-up tilt that would have activated otolith and body tilt receptors (23, 64). Habituation with head-up tilt takes significant time in the upright position and, therefore, would not involve the vertical semicircular canals. Whether this would make their habituating stimulus less powerful then sGVS, which activates the combined input from the vertical semicircular canals and the otolith organs to the vestibular nuclei (27, 28) is not known. Why these findings were not supported by the studies of Foglia-Manzillo (30) and Duygu (31) is also not clear. Perhaps as suggested by Foglia-Manzillo, the use of the boring bouts of static head-up tilt that formed the basis of the static tilt treatment was responsible for non-compliance among the subjects.

Sinusoidal galvanic vestibular stimulation (sGVS) has more potential for use in this regard. It has been widely used to activate MSNA without serious consequences other than production of motion sickness in some of the test subjects (37–40, 65–67). The apparatus to deliver sGVS is small, electrically safe, and the electrodes are easy to apply over the mastoids. During such habituation, moreover, it would be possible for subjects to read, listen to music, and/or watch TV or a movie during the habituation periods.

Although relatively few rats were intensively studied, it was also possible to gain a preliminary estimate of the relative strength of the various stimuli on the otolithic system from a comparison of the effects of the nose-up tilts and sGVS on the changes in BP and HR during development of habituation (Figures 2 and 3). The ±2 and ±3 mA sGVS were more potent in causing continued dissociation of BP and HR than the nose-up tilts that imposed a known load of ≈0.91 *g* on the head and body (Figures 2 and 3). This implies that the sGVS was perceived by the vestibular system as somewhat more potent than the nose-up tilts that were equivalent to 0.91 *g*. We can estimate, therefore, that the sGVS was probably perceived as being equivalent to about 1 *g*, which is a similar estimate of the strength of the sGVS that we previously derived from a comparison of its effect on the low-frequency activity in BP and HR (33).

### Limits of the Study

Although the results were clear cut in the four rats that were intensively studied in this report and in the seven rats used in previous studies, additional rats must be used to determine how long habituation is maintained, and whether, in accordance with the definition of Rankin et al. (1) and Thompson (2), reactivation of the habituated state would be considerably faster than the original habituation process. More rats are also needed to determine whether ±2 mA sGVS is as effective in producing habituation as ±3 mA sGVS, since there have been a wide range of studies using ±2 mA sGVS, which is safe and without significant side effects in humans. Nevertheless, despite the limited number of animals, the results were deemed to be of potential value in directing habituation of syncope in humans.

A critical question is what is stimulated by the sGVS. Galvanic stimuli activate all axons in the vestibular nerves (68, 69). This causes excitation of canal and otolith neurons in the Vestibular Nuclei related to rotation and tilt. The lateral canal-related neurons are rapidly inhibited, however, leaving the otolith-related input intact (26, 70). The sGVS causes generation of cfos in otolithic portions of the vestibular nuclei and in the superior vestibular nuclei (27), suggesting that the vertical canal input may also be maintained. Neurons in these nuclei project monosynaptically through the inferior vestibular nuclei to the rostral ventral lateral medulla bilaterally. Utilizing glutamatergic transmission (27–29, 71), they excite paraspinal neurons in the sympathetic chain in the thoracic spinal cord (72) to produce the VSR, probably through constriction of peripheral vessels because the VSR does not cause significant changes in HR (35).

An alternate pathway could also have been activated by the sGVS, namely a branch of the vagus nerve that originates in the tragus of the external ear and projects to the NTS and the dorsal motor nucleus of the vagus (73). Therefore stimulation of the tragal branch of the vagus could have affected the function of the NTS, namely its control of the baroreflex, possibly causing modification of the baroreflex sensitivity that was observed with continued stimulation. However, there was also a response to 70° nose-up tilt that would not have activated the vagus nerve endings in the tragus. In fact, 60° and 70°Head-up tilts are widely used to determine if humans suspected of having vasovagal syncope become faint during the tilt-test, and small currents of galvanic vestibular stimulation also maintain BP during head-up tilts (74). Moreover, the sequence of habituation was characterized by an increase in HR to counter the drop in BP, and activation of the vagus would cause a reduction in HR, not an increase. Therefore, it is not likely that vagus activation from the tragus had significantly affected the results shown in this study.

Before such use in humans can be considered, however, it is essential to determine that the bouts of habituation do not cause serious side effects, and to determine how long the habituation produced by such an apparatus persists, and how often the treatments have to be repeated to maintain the habituation. It would also be critical to know the origin or the nature of the signal that causes the baroreflex to come alive in the anesthetized rat. Neither do we know the duration of the habituation process that is generated by activation of the VSR. Nevertheless, this work provides support for studies that suggest that vasovagal syncope can be habituated. For the first time, it demonstrates that habituation of vasovagal oscillations and vasovagal responses, the underlying processes that lead to vasovagal or neurogenic syncope, can be blocked by repetitive activation of the VSR. Furthermore, it indicates that sGVS with ±2 or ±3 mA, 0.025 Hz is likely to be a strong habituating stimulus in humans as in rats.

## Author Contributions

BC conceived the study, monitored its progress, and wrote the paper. GM helped to plan the study, did the surgery, monitored care of the animals, and made critical suggestions to improve the manuscript. SY helped to plan the study, performed the experiments, analyzed the data, made the figures, and helped with the manuscript. YX analyzed the data and did the analysis of low-frequency activity and baroreflex sensitivity. TR initiated and supervised the wavelet analyses and the results of baroreflex sensitivity.

## Conflict of Interest Statement

The authors declare that the research was conducted in the absence of any commercial or financial relationships that could be construed as a potential conflict of interest.

## Abbreviations

BP, arterial blood pressure; bpm, heart in beats per minute; CVLM, caudal ventral lateral medulla; *g*, imputed acceleration of gravity; HR, heart rate; MSNA, muscle sympathetic nerve activity; NTS, nucleus tractus solitarius; RVLM, rostral ventral lateral medulla; sGVS, sinusoidal Galvanic Vestibular Stimulation; VSR, vestibulosympathetic reflex; ≈, approximate; °, degrees.
